# Microbial network structure, not plant and microbial community diversity, regulates multifunctionality under increased precipitation in a cold steppe

**DOI:** 10.3389/fmicb.2023.1349747

**Published:** 2024-01-12

**Authors:** Xuechen Yang, Wenzheng Song, Xue Yang, Tianxue Yang, Wenqing Bao, Chengliang Wang, Junqin Li, Shangzhi Zhong, Qi Jiang, Lu-Jun Li, Wei Sun

**Affiliations:** ^1^State Key Laboratory of Black Soils Conservation and Utilization, Northeast Institute of Geography and Agroecology, Chinese Academy of Sciences, Harbin, China; ^2^Institute of Grassland Science, Key Laboratory of Vegetation Ecology of the Ministry of Education, Jilin Songnen Grassland Ecosystem National Observation and Research Station, Northeast Normal University, Changchun, China; ^3^School of Civil Engineering and Transportation, Northeast Forestry University, Harbin, China; ^4^Grassland Agri-Husbandry Research Center, College of Grassland Science, Qingdao Agricultural University, Qingdao, China; ^5^No. Fifteen Senior High School of Mudanjiang, Mudanjiang, China

**Keywords:** biodiversity, co-occurrence ecological networks, ecosystem function, grassland ecosystem, nutrient cycling, rainfall amount

## Abstract

It is known that the dynamics of multiple ecosystem functions (i. e., multifunctionality) are positively associated with microbial diversity and/or biodiversity. However, how the relationship between microbial species affects ecosystem multifunctionality remains unclear, especially in the case of changes in precipitation patterns. To explore the contribution of biodiversity and microbial co-occurrence networks to multifunctionality, we used rainfall shelters to simulate precipitation enhancement in a cold steppe in Northeast China over two consecutive growing seasons. We showed that an increased 50% precipitation profoundly reduced bacterial diversity and multidiversity, while inter-annual differences in precipitation did not shift microbial diversity, plant diversity, or multidiversity. Our analyses also revealed that increased annual precipitation significantly increased ecosystem, soil, nitrogen, and phosphorous cycle multifunctionality. Neither increased precipitation nor inter-annual differences in precipitation had a significant effect on carbon cycle multifunctionality, probably due to the relatively short period (2 years) of our experiment. The co-occurrence network of bacterial and fungal communities was the most dominant factor affecting multifunctionality, the numbers of negative interactions but not positive interactions were linked to multifunctionality. In particular, our results provided evidence that microbial network topological features are crucial for maintaining ecosystem functions in grassland ecosystems, which should be considered in related studies to accurately predict the responses of ecosystem multifunctionality to predicted changes in precipitation patterns.

## 1 Introduction

Precipitation is an important factor in controlling biodiversity and ecosystem function, especially in semiarid and arid ecosystems. Grasslands have experienced dramatic shifts in structure and function driven by changes in precipitation patterns (Bai et al., 2008, 2012). A large body of research has confirmed that changes in precipitation could alter the organism's community composition and structure, thereby regulating grassland productivity and ecosystem function, such as the cycling of energy and nutrients (Nielsen and Ball, 2015; Ochoa-Hueso et al., 2018; Fahey et al., 2020). However, most previous studies have only addressed individual ecosystem functions in response to precipitation (Delgado-Baquerizo et al., 2014; Li et al., 2023). We also need to assess whether precipitation change can alter multiple functions simultaneously (i.e., multifunctionality).

Multifunctionality, as an integrative manifestation of multiple ecological functions, can reflect the overall functionality, services, and processes (e.g., including primary production, nutrient cycling, organic matter decomposition, carbon [C] storage, and greenhouse emissions), which provides an insight into predicting the effect of climate change on a multi-temporal and spatial scale (Hu et al., 2022; Xue et al., 2023). Recent investigation has revealed that multifunctionality was profoundly reduced by decreased precipitation, but increased precipitation had less impact on multifunctionality (Zhai et al., 2024). Guo et al. (2023) have shown that mean annual precipitation (MAP) could affect multifunctionality by changing the plant species' alpha and functional beta diversity. Additionally, multifunctionality was also weakened by decreased precipitation frequency in a degraded Horqin grassland, and ecosystem coupling had a strong direct positive effect on multifunctionality (Yang et al., 2023). Despite the fact that changes in precipitation can directly or indirectly affect multifunctionality, few studies have explored the response of multifunctionality to increased precipitation under inter-annual variation in precipitation. Yet, it remains unclear which mediating variable is the better predictor of multifunctionality.

Biodiversity is the foundation for maintaining ecosystem functions, and it usually has a positive effect on multifunctionality (Bardgett and van der Putten, 2014; Anujan et al., 2021; Guo et al., 2021). For example, plant species diversity acted as a predictor that could enhance multifunctionality in global drylands (Maestre et al., 2012). Another global study found that the effect of plant diversity on multifunctionality was indirect and mediated by soil biodiversity (Delgado-Baquerizo et al., 2020). Soil biodiversity plays an essential role in supporting the sustainable productivity of terrestrial ecosystems (Han et al., 2021). Particularly, soil microbes are the most diverse and abundant organisms in soils that are strongly related to multifunctionality. Our previous research showed that increased annual precipitation shifted the dominant microbes from the bacterial community to the fungal community (Yang et al., 2021), and bacterial biomass directly explained the ecosystem, C, and nitrogen (N) cycle multifunctionality to inter-annual precipitation change (Cui et al., 2020). However, soil microbes comprise a vast diversity and abundance of different microbial groups and complex trophic interactions (Bardgett and van der Putten, 2014; Banerjee et al., 2018). In recent years, microbial co-occurrence network analysis has provided a statistical tool to explore the interactions in a range of environments, providing important details on microbial cooperation and competition (Chen et al., 2020). Hu et al. (2021) reported that a more stable microbial network could promote crop growth and yields in a rotation system. Moreover, an 8-year soil plot transplantation experiment has indicated that MAP could influence C sequestration in glomalin and soil aggregates by regulating the diversity and network complexity of arbuscular mycorrhizal fungi (Yang et al., 2022). The clustering coefficient of the fungal network rather than the bacterial network was the key factor predicting the soil C metabolic profile (Xiao et al., 2018). Numerous previous studies have been conducted on how microbial network complexity responded to climate change and subsequently affected individual ecosystem functions. At present, the major challenge is to link microbial co-occurrences to processes that contribute to multifunctionality under precipitation patterns change.

In this study, we conducted a 2-year field experiment in a cold steppe in Northeast China to evaluate the increased precipitation effects on plant and microbial diversity, microbial network structure, and multifunctionality. Multifunctionality refers to multiple ecosystem functions, including plant community leaf C and N content, bacterial and fungal biomass, soil dissolved organic C (DOC) content, net and potential N mineralization rate, ammonia-oxidizing archaea (AOA) and bacteria (AOB) abundance, soil available phosphorus (AP) content, and alkaline phosphatase (ALP) activity, which are essential for sustainable managed ecosystem functioning by supporting key ecosystem services such as primary production and organic matter decomposition (Table 1). We hypothesized that (1) the response of multifunctionality to increased precipitation is dependent more on the annual and ambient precipitation, and (2) microbial species interactions (network structure) than biodiversity and/or multidiversity, which would be the primary factors driving multifunctionality.

**Table 1:** Ecosystem functions and their corresponding indicators.

**Ecosystem indicators**	**Biogeochemical cycle**	**Functions**
**Above-ground**		
Plant community leaf C	C cycle	Limit primary production
Plant community leaf N	N cycle	Supply N sources
**Below-ground**		
Bacterial biomass	C cycle	Support below-ground functionality
Fungal biomass	C cycle	Support below-ground functionality
Soil dissolved organic C content	C cycle	Decomposition of organic C
Net N mineralization rate	N cycle	Organic N mineralization
Potential N mineralization rate	N cycle	Organic N mineralization
Ammonia-oxidizing archaea abundance	N cycle	Catalytic ammonia oxidation
Ammonia-oxidizing bacteria abundance	N cycle	Catalytic ammonia oxidation
Soil available P content	P cycle	Provide available P
Alkaline phosphatase	P cycle	Organic P mineralization

## 2 Materials and methods

### 2.1 Site description

The study was conducted at the Jilin Songnen Grassland Ecosystem National Observation and Research Station of Northeast Normal University (44°40′N, 123°45′E, 160 m), Northeast China, in 2016–2017. The climate is classified as semiarid continental monsoon, and annual precipitation varies between 258 and 716 mm (1953–2017), with 75% falling during the growing season, especially between June and August. The precipitation coefficient of variance (CV) is close to 26%. The annual temperature ranges from 3.4°C to 7.6°C (Zhu et al., 2019; Li et al., 2023). The site consists of sodic saline meadow soil, and the soil is alkaline (pH 9.16), total carbon (TC) 8.96 g kg^−1^, total nitrogen (TN) 0.84 g kg^−1^, and total phosphorous (TP) 0.04 g kg^−1^. Plant communities are dominated by the perennial grass *Leymus chinensis*, accompanied by other annual or perennial herbs, such as *Hemarthria altissima, Phragmites australis*, and *Chloris virgata*.

### 2.2 Experimental design

We fenced 1 ha (100 × 100 m) of grassland in August 2015, and the experiment was laid out in a randomized complete block design. Four blocks with similar vegetation compositions were established within the fenced grassland. Each block had five plots (3.5 m × 3.5 m; buffer zones were at least 1 m wide between the adjacent plots), which were randomly assigned to the five levels of precipitation: decreased 50% (D50), decreased 30% (D30), ambient (I0), increased 30% (I30), and increased 50% (I50). Each plot was surrounded by iron sheets (0.15 m above-ground and 0.5 m below-ground) to isolate the plot from overland runoff and below-ground lateral soil infiltration. Precipitation treatments were initiated using rainfall shelters throughout the growing season, the details are shown in Yang et al. (2021). After each rainfall event, we collected rainfall in the D30 and D50 plots and added it to the I30 and I50 plots, respectively. The rainfall collected in the I0 (providing a 30% rainfall decrease), I30, and I50 plots were added back to the corresponding plots.

Air temperature and daily precipitation were measured using an RG2-M sensor (Onset Computer Corporation, Bourne, MA, United States). In the study site, the growing season precipitation was 204 mm in 2016 and 398 mm in 2017 (from April to August sampling dates). Considering long-term mean precipitation from April to early August (241 mm), we defined 2016 as the dry year and 2017 as the wet year (Yang et al., 2021). Soil moisture and temperature (30 cm depth) monitoring sensors (CR200 Datalogger, Campbell Scientific Inc., Logan, UT, United States) were installed in a representative block, and they were measured at 30-min intervals (Supplementary Figure S1).

### 2.3 Vegetation and soil sampling

Vegetation surveys and soil sampling were conducted in August 2016 and 2017. In each plot, we surveyed the number of plant species and accounted for the individual numbers of each species in a permanently placed quadrat (0.5 m × 1 m). Plant community diversity was calculated as the Shannon-Wiener index. Following the vegetation survey, 10 healthy and green plant leaf samples were selected in each plot and oven-dried for 48 h at 65°C, and then analyzed for leaf total C and N concentration with an elemental analyzer (vario EL cube, Elementar, Langenselbold, Germany) after grinding with a Wiley ball mill (MM400, Retsch, Hanau, Germany).

Five soil cores (0–15 cm depth) within the center of each plot (2.5 m × 2.5 m) were randomly taken using an auger (ϕ 25 mm). These five subsamples were mixed and sieved with stainless steel mesh filters (mesh size 2 mm) to obtain one composite sample. Soil samples were transported to the laboratory and separated into three fractions. The first fraction was stored at −20°C until the microbial biomass, diversity, and functional gene abundance could be analyzed. The second fraction was stored at 4°C for the analysis of soil DOC content, net N mineralization rate, potential N mineralization rate, and ALP activity within 1 week. The third fraction was air-dried for 1 month in order to analyze the soil AP content.

### 2.4 Soil function parameters measures

Soil DOC was extracted using 0.05 M potassium sulfate and determined using the dichromate oxidation method (Vance et al., 1987). A quantity of 10 g of fresh soil samples was rewetted to reach 80% of field water holding capacity and incubated in the dark for 2 weeks at 30°C. The net N mineralization rate was calculated as the accumulation of total inorganic N over the course of the anaerobic incubation (Delgado-Baquerizo et al., 2014). We measured the potential N mineralization rate using the chloroform fumigation-incubation method (Durán et al., 2013). The fumigated sample is inoculated with fresh soil, and microbes from the fresh soil grow vigorously using the killed cells from the fumigated soil as substrate. The soils were then incubated for 10 days, and inorganic N was released during the incubation. The potential N mineralization rate was calculated as the accumulation of total inorganic N over the course of the incubation. Inorganic N was extracted by 2 M potassium chloride and determined by a continuous flow analytical system (Alliance Flow Analyser, Futura, Frépillon, France). Soil AP was extracted with sodium bicarbonate and then determined using the molybdenum-blue method. ALP activity was determined by labeling the fluorescent substrate, as reported by Bååth and Anderson (2003) and Trivedi et al. (2016). Fluorescence values were determined using a plate fluorescence reader (TECAN Infinite F200, Tecan Group, Switzerland) with excitation and emission filters of 365 nm and 450 nm, respectively.

We used phospholipid fatty acids (PLFAs) to characterize microbial biomass (Frostegård et al., 2011). PLFA analysis was performed with a gas chromatograph (Agilent 6890, Agilent Technologies, Palo Alto, United States) and the MIDI Sherlock Microbial Identification System (MIDI Inc., Newark, United States). The bacteria included PLFAs i14:0, 15:0, i15:0, a15:0, i16:0, 16:1ω7c, i17:0, a17:0, cy17:0, 17:1ω8c, 10Me17:0, and 18:1ω7c; fungi included PLFAs 16:1ω5c and 18:1ω9c. The prefixes “i”, “a”, “Me”, and “cy” represent iso, anteiso, mid-chain methyl branching, and cyclopropyl rings, respectively (Dai et al., 2023).

### 2.5 DNA extraction, (q)PCR, sequencing, and bioinformatics analysis

Soil DNA was extracted using the MO BIO PowerSoil™ DNA Isolation Kit (MO BIO Laboratories, Inc., Carlsbad, CA, United States). DNA concentrations were quantified with a NanoDrop 2000 spectrophotometer (Thermo Fisher Scientific Inc., Waltham, MA, United States). The quality of the DNA extracts was checked by 1% agarose gel electrophoresis and stored at −80°C for further analysis.

Quantitative PCR was performed on a real-time detection system (Light Cycler 480 Software Setup, Roche Molecular Systems, Inc., Pleasanton, CA, United States) to quantify the abundances of AOA and AOB *amoA* genes. The pair primers for these functional genes are shown in Supplementary Table S1. The total volume (20 μl) of the reaction systems contained 10 μl SybrGreen qPCR Master Mix, 0.4 μl forward and 0.4 μl reverse primer, 2 μl amplification template (genomic DNA), and 7.2 μl sterile ddH_2_O. The thermal cycling programs were as follows: 95°C for 3 min, 50 × (95°C, 15 s; 57°C, 20 s; and 72°C, 30 s), and 72°C for 5 min. Standard curves for both genes were generated by preparing standards from purified PCR products amplified by each specific set of primers. The copy number was calculated based on the size of the fragment (Fogel et al., 1999). Standards were run on each qPCR plate, and linear standard curves were used to estimate copy numbers.

The V4 hypervariable gene region of the bacterial 16S rRNA and the fungal ITS1 gene region were subjected to high-throughput sequencing by Novogene Co., Ltd. (Beijing, China) using the Illumina Hiseq 2500 sequencing platform (Illumina, Inc., San Diego, CA, United States). Raw sequences were demultiplexed, quality-filtered by fastp, and merged using FLASH. Operational taxonomic units (OTUs) were clustered at the 97% similarity level using UPARSE (Yang et al., 2021). The SILVA and UNITE databases were used with a confidence threshold of 80% to analyze the taxonomy of representative sequences of bacteria and fungi. OTU abundance information was normalized via a standard of the sample with the least sequence number. Shannon-Wiener diversity was evaluated using the MOTHUR software.

Microbial co-occurrence network analyses based on Spearman's rank were performed using abundance data on the genus level of bacterial and fungal communities. The co-occurrence patterns were explored based on a strong correlation with *P* < 0.01 and Spearman's ρ > |0.8| (Wu et al., 2021). The nodes in networks represented OTUs, whereas the edges corresponded to a strong and significant correlation between nodes. Modularity is an index measuring the extent to which a network is divided into modules, and >0.4 was used as the threshold to define modular structures (Newman, 2006). The topological coefficients of each plot subnetwork, including the number of edges, average degree, average path length, density, and clustering coefficient, were calculated to estimate the complexity of the network (Xu et al., 2023).

### 2.6 Assessing multidiversity and multifunctionality

We combined the biodiversity characteristics (soil bacterial, fungal, and plant diversity) by averaging the standardized scores (min-max normalization) of diversity to obtain a single index reflecting the multidiversity (Zhang et al., 2023). We used z-transformation to normalize and standardize each of the 11 ecosystem functions to generate a quantitative ecosystem multifunctionality index. The index was created by averaging the standardized ecosystem functions (Delgado-Baquerizo et al., 2016). The same method was used to obtain the soil, C, N, and P cycle multifunctionality.

### 2.7 Statistical analysis

Statistical analysis was conducted in R version 3.6.3 (R Development Core Team). A linear mixed-effect model was used to examine the effects of increased precipitation, inter-annual variations in precipitation (year), and their interaction on plant diversity, microbial diversity, microbial network properties, C, N, P cycling function parameters, and multifunctionality. Increased precipitation and year were treated as fixed factors, and plots nested into blocks were treated as random factors. Bonferroni's *post-hoc* test was used in the case of significant treatment interactions. All data were assessed for homogeneity and log-transformed when necessary. The significance level was set at *P* < 0.05. Graphs were drawn using SigmaPlot, and the microbial networks were visualized by Gephi. The Mantel test and Spearman correlation analysis were used to determine the main factors affecting multifunctionality using the R package “linkET.”

## 3 Results

### 3.1 Biodiversity and microbial network properties

**Figure 1:**
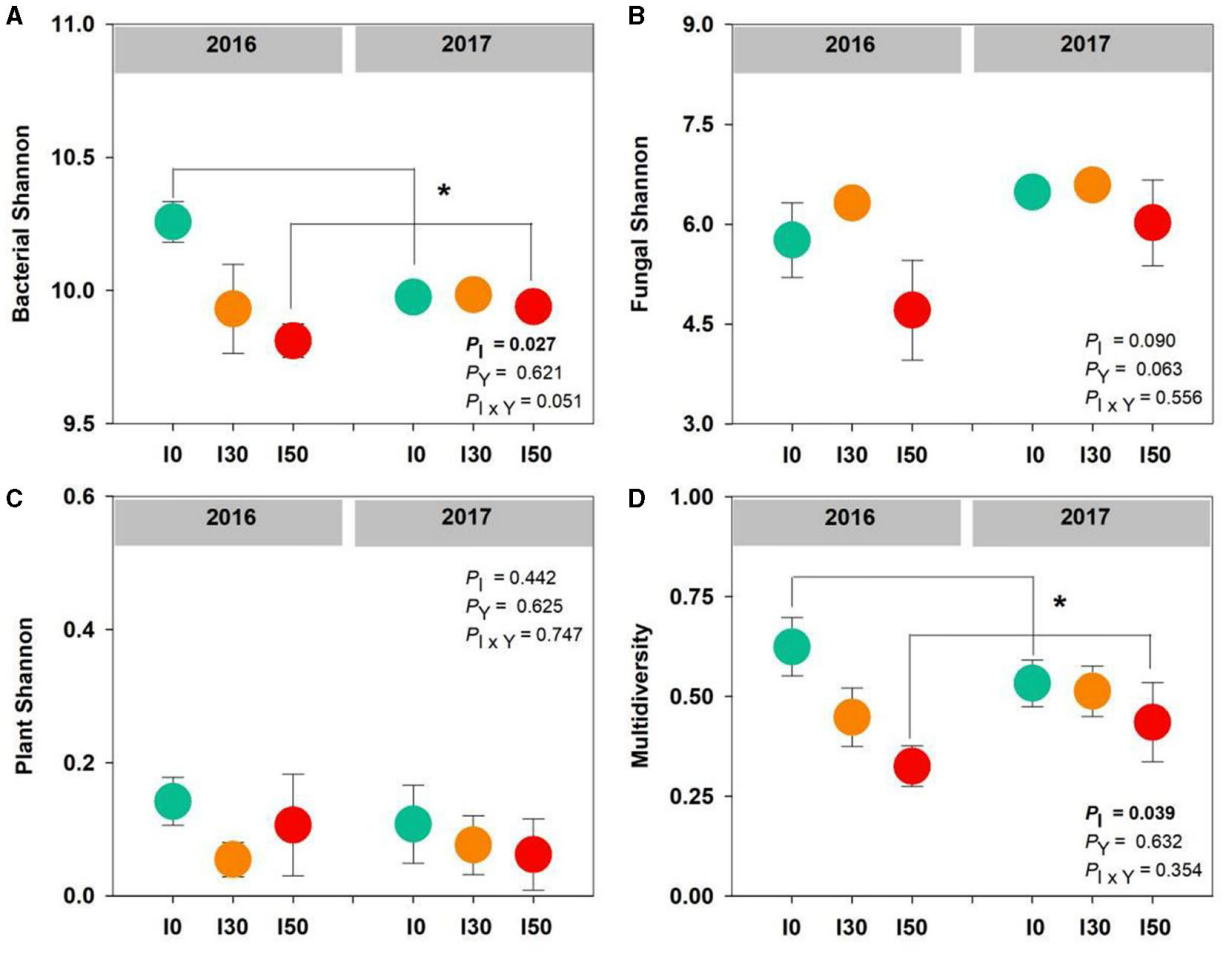
The effects of increased precipitation on bacterial diversity **(A)**, fungal diversity **(B)**, plant diversity **(C)**, and multidiversity **(D)** in 2016 and 2017. Significant differences are indicated by *(*P* < 0.05). Error bars represent ± SE.

We found a significant effect of increased precipitation on bacterial diversity and multidiversity; compared to the I0 plots, they decreased by 2% and 34% at the I50 plots, respectively (both *P* < 0.05; Figures 1A, D). However, inter-annual differences and the interactive effects of increased precipitation and inter-annual differences had no significant effects on bacterial, fungal, plant diversity, or multidiversity (all *P* > 0.05; Figure 1).

**Figure 2:**
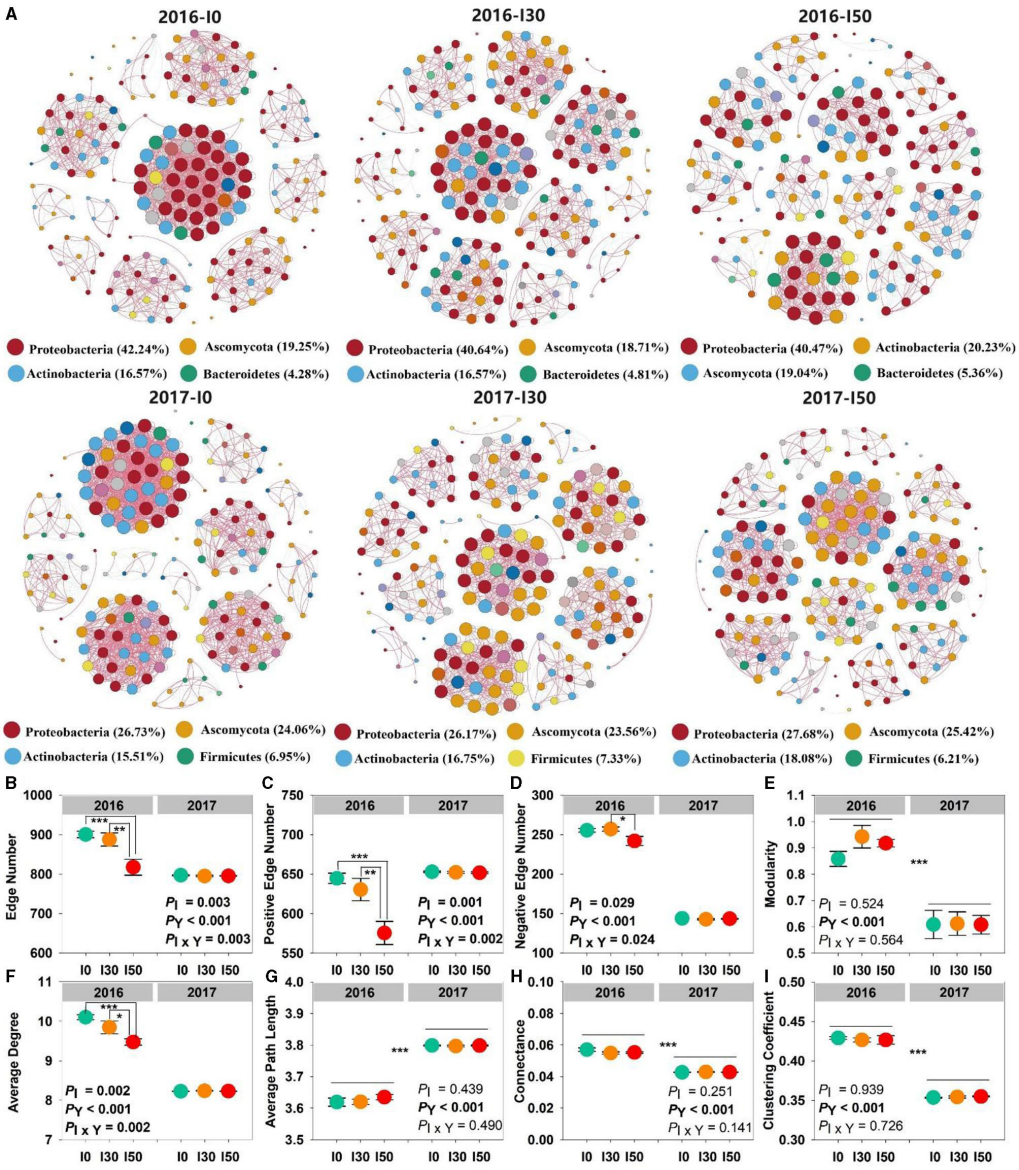
Changes in the complexity of microbial networks under increased precipitation in 2016 and 2017. Connections in the network **(A)** indicate strong and significant correlations. The network complexity is calculated by extracting the eight topological coefficients **(B–I)** of the sub-network. Significant differences are indicated by *(*P* < 0.05), **(*P* < 0.01), and ***(*P* < 0.001). Error bars represent ± SE.

Microbial network analysis showed distinct co-occurrences of bacterial and fungal members. The microbial community was dominated by the phyla Proteobacteria, Actinobacteria, and Ascomycota in both experimental years. However, in the wet year (2017), phyla Firmicutes (6%−7%) replaced the dominance of Bacteroidetes (4%−5%) that was in the dry year (2016). We found that the network complexity (interactions) within bacterial and/or fungal communities was decreased by increased precipitation treatment. Complexity was measured as the number of edges, and they decreased with the increased precipitation gradient in both years (Figure 2A). The majority of network interactions were positive, with more than 550 edges, and the negative edges were <300. Significant main and interactive effects of increased precipitation and inter-annual differences on the edge, positive edge, negative edge number, and average degree were observed (all *P* < 0.05). In 2016, the lowest values were detected at I50 plots (817.5 ± 20.1, 575.5 ± 14.7, 242 ± 5.8, 9.5 ± 0.1, respectively; Figures 2B, D, F). The values of modularity, average path length, connectance, and clustering coefficient had significant inter-annual differences (all *P* < 0.001; Figures 2E, G–I).

### 3.2 Individual ecosystem functions and multifunctionality

Significantly main and interactive effects of increased precipitation and inter-annual differences on plant leaf C content were detected. Increased precipitation significantly shifts plant leaf N content (*P* < 0.05), the highest and lowest values were observed in the I30 and I50 plots in both years. Soil fungal biomass (*P* < 0.01), potential N mineralization rate, AOA abundance, AP content, and ALP activity (all *P* < 0.001) had significant inter-annual differences, which in the dry year were significantly lower than those in the wet year. No significant main and interactive effects of increased precipitation and inter-annual differences were observed on soil bacterial biomass, DOC content, net N mineralization rate, and AOB abundance (all *P* > 0.05; Supplementary Tables S2, S3).

**Figure 3:**
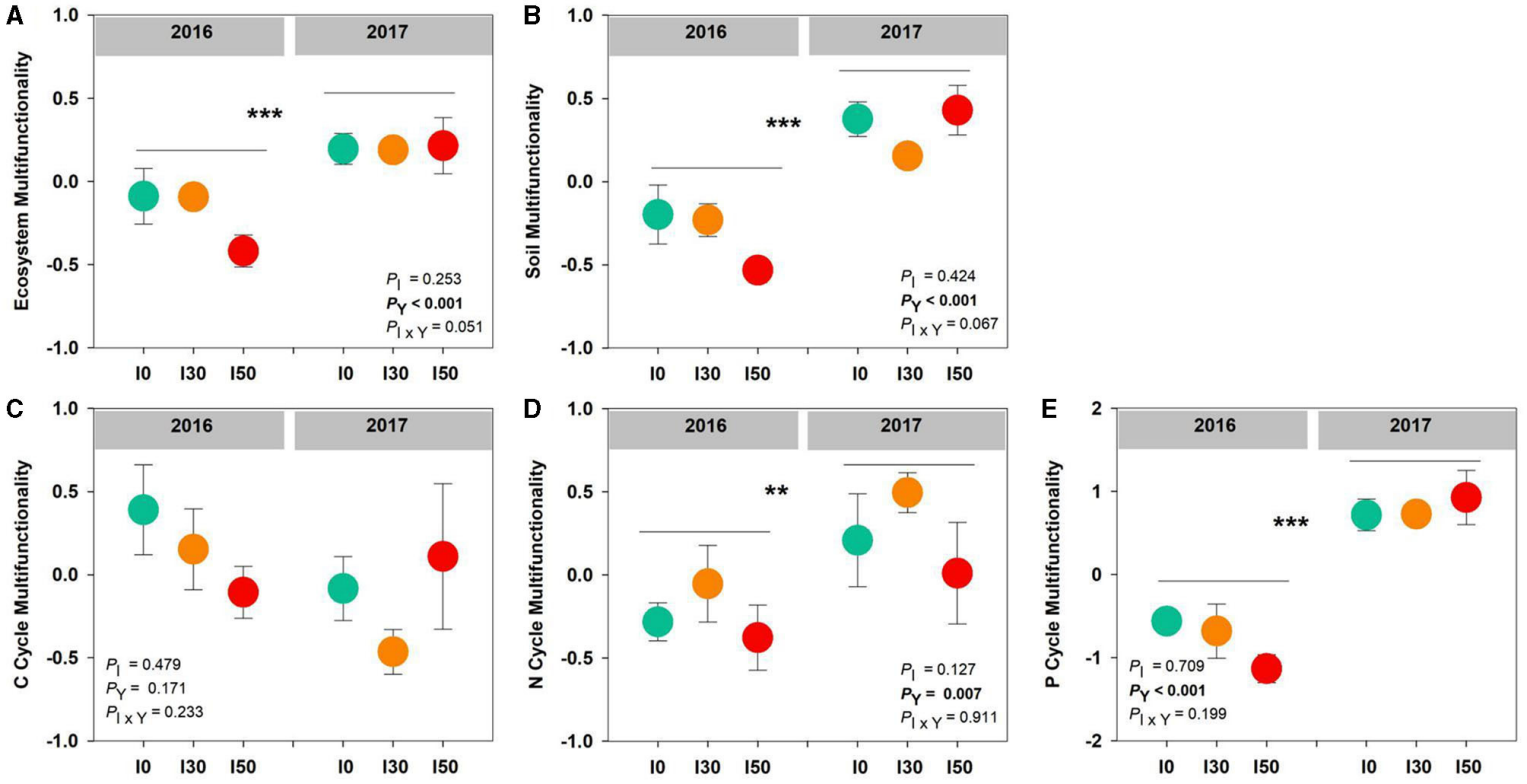
The effects of increased precipitation on the ecosystem **(A)**, soil **(B)**, C cycle **(C)**, N cycle **(D)**, and P cycle **(E)** multifunctionality in 2016 and 2017. Significant differences are indicated by **(*P* < 0.01) and ***(*P* < 0.001). Error bars represent ± SE.

Increased precipitation did not significantly alter ecosystem, soil, C, N, and P cycle multifunctionality (all *P* > 0.05). However, ecosystem, soil, N, and P cycle multifunctionality had significant inter-annual differences (all *P* < 0.001), the average values were lower in 2016 than those in 2017. No significant interactive effects between increased precipitation and inter-annual differences were detected on all multifunctionalities (all *P* > 0.05; Figure 3).

### 3.3 Relationships among biodiversity, microbial networks, and multifunctionality

**Figure 4:**
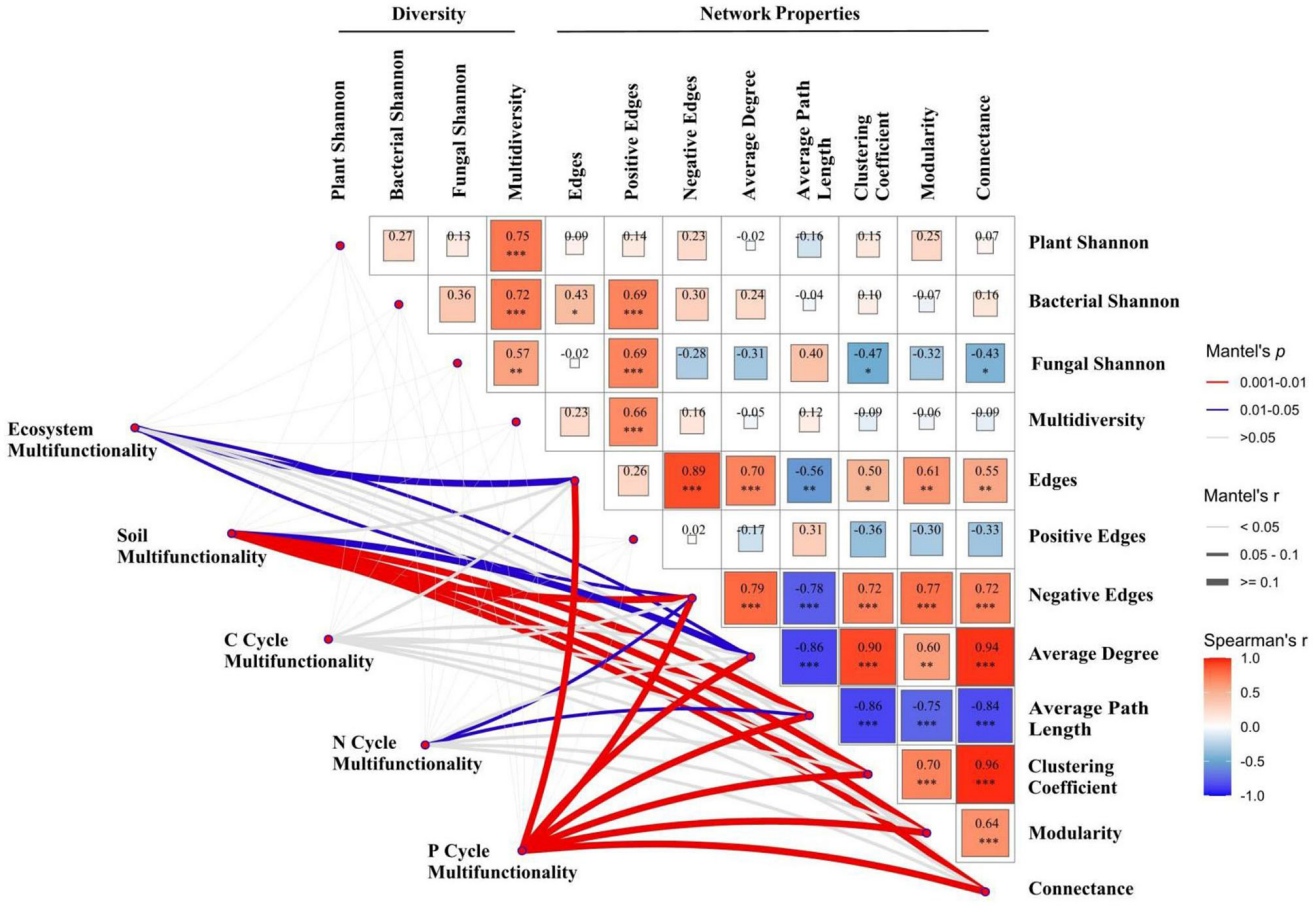
Correlation analysis of biodiversity and microbial network properties, and the Mantel test analysis of multifunctionality, biodiversity, and microbial network properties in 2016 and 2017. Significant differences are indicated by *(*P* < 0.05), **(*P* < 0.01), and ***(*P* < 0.001).

The Mantel test and Spearman correlation analysis indicated that ecosystem multifunctionality was significantly correlated with microbial network edges and negative edges (both *P* < 0.05). Soil multifunctionality is significantly related to average degree (*P* < 0.05), average path length, clustering coefficient, modularity, and connectance (all *P* < 0.01). Neither biodiversity nor microbial network properties could explain the variations in C cycle multifunctionality (*P* > 0.05). Negative edges and average path length explained the largest fraction of the variation in N cycle multifunctionality (*P* < 0.05). Microbial network edges, negative edges, average degree, average path length, clustering coefficient, modularity, and connectance explained the largest statistically significant correlation in P cycle multifunctionality (all *P* < 0.01; Figure 4).

## 4 Discussion

### 4.1 Effect of changes in precipitation on biodiversity

Changes in precipitation can alter the diversity and composition of the soil microbial community, and microbial community diversity always increases with increasing precipitation (Cregger et al., 2012). Unexpectedly, we found a significant decline in bacterial Shannon diversity in association with increased precipitation (Figure 1A). The previous study reported that soil microbes have an optimal precipitation range for diversity (Yang et al., 2021). As the natural precipitation CV stands to 26% at our studied grassland, we believed that the increased 50% precipitation had exceeded the optimal range for bacteria, resulting in a reduction in bacterial diversity. In contrast to bacteria, fungi diversity was not sensitive to precipitation change or current soil moisture change (Figure 1B); this was consistent with the previous result of Yang et al. (2021). Because fungi were more resistant than bacteria to water stress, both the immediate and delayed changes in soil moisture affected fungi less than they did bacteria (de Vries et al., 2018).

Microbial co-occurrence network structure was determined by both increased precipitation and inter-annual differences in precipitation, and increased precipitation significantly reduced interactions within the microbial community (Figures 2B–D). The network changes indicated that the interactions, at least the potential to affect each other among bacterial and/or fungal OTUs, can be feebler or more intense in precipitation change (He et al., 2017). The inter species relationships (e.g., synergy, competition) between different bacterial and/or fungal species were weakened by increased precipitation, especially in the dry year. It was widely accepted that the positive interactions could be related to cross-feeding and niche overlap within the community, whereas the negative interactions were attributed to competition and amensalism (Faust and Raes, 2012). In this study, the number of positive edges in all networks was greater than the number of negative edges, indicating more microbial community cooperation than competition. The co-occurring species generally share similar ecological niches and life-history strategies, and they are often more adaptable to homogeneous habitats. This result was consistent with Yang et al. (2022). Moreover, modularity can be considered a strategy to maintain the stability of the soil microbial community, resulting in faster and better adaptation of microbial species to environmental changes (Chen et al., 2020). The lower modularity in the wet year (Figure 2E) indicated that an extreme one-time rainfall event (203 mm in early August) reduced microbial community stability, it may be explained by a decline in microbial activity under waterlogging (Yang et al., 2021).

Unlike microbes, plant Shannon diversity was unchanged by precipitation change (Figure 1C). The studied grassland was dominated by *L. chinensis*, which contributed more than 85% of plant cover before the initiation of the increased precipitation treatment in 2015. Our experimental treatment lasted only 2 years, which did not induce significant changes in plant species diversity. The responses of plants and microbes to increased precipitation were asynchronous, the bacterial response was quick, whereas the plant response was relatively slow. However, we found that the response of multidiversity to precipitation change was highly consistent with the bacterial diversity response (Figure 1D), suggesting that multidiversity was dependent more on bacterial diversity than plant diversity.

### 4.2 Effect of changes in precipitation on ecosystem functions

There were significant responses of the plant community leaf C content to increased precipitation, inter-annual precipitation, and their interactions (Supplementary Tables S1, S2), although no shift in plant diversity was observed. These were similar to the results of a previous experiment in Hulunber grassland subjected to increased precipitation (Ma et al., 2012). Several studies showed that plant leaf C was usually positively correlated with plant height and biomass (Legay et al., 2014; Adair et al., 2019), suggesting that plant growth and reproduction were limited by water availability in this semiarid grassland. In addition, we found that the increased 50% precipitation had the lowest plant community leaf N content, especially in the wet year. The wetter soil was conducive to denitrification, and a high baseline of N_2_O emissions was probably the main factor driving the low N uptake capacity of *L. chinensis* (Shi et al., 2021).

No effects of increased precipitation on individual soil functions have been reported in the present study. However, fungal biomass (FB), potential N mineralization rate, AOA abundance, AP content, and ALP activity were positively correlated with increased annual precipitation. The response of FB was in line with the previous finding that fungi were highly responsive to changes in inter-annual precipitation (Yang et al., 2021), and the amplified microbial populations could stimulate soil organic matter mineralization and nitrification. According to Chen et al. (2017), the AOA community could be constrained by substrate availability when competing with other microbes. We found that AOA microbes in the wet year were more abundant than their bacterial counterpart, indicating that soil N availability was sufficiently high under increased annual precipitation. For the soil P cycle functions, the accumulations of microbial biomass in the wet year might increase the demand for soil P availability, which resulted in multiple changes in the accelerating P cycle associated with increased soil organic P mineralization rate, further promoting AP and ALP (Cui et al., 2021).

Precipitation is an important limiting factor for soil nutrients, plant growth, and vegetation development in terrestrial ecosystems (Guo et al., 2023). Consistent with our first hypothesis, inter-annual variations in precipitation, rather than increased precipitation, strongly influence ecosystem, soil, N, and P cycle multifunctionality (Figure 3). A 5-year experiment conducted in three Inner Mongolian temperate steppes also demonstrated that multifunctionality was not more sensitive to increased precipitation, even in the desert (Zhai et al., 2024). The promotion of individual functions induced by increased annual precipitation further led to higher multifunctionality (Yang et al., 2023). It could be explained by the fact that higher annual precipitation can enhance soil nutrient availability by accelerating organic matter decomposition and mineralization (Hu et al., 2022), resulting in increases in ecosystem functions.

### 4.3 Microbial co-occurrence network pattern drives multifunctionality

Many studies have investigated the positive effects of biodiversity and/or multidiversity on multifunctionality under changes in precipitation patterns (Dacal et al., 2022; Hu et al., 2022). On the one hand, plant diversity can improve multifunctionality through complementary resource use and facilitation between plant species (Guo et al., 2023). On the other hand, microbial diversity can support multifunctionality in broader ways, they carry out critical ecological processes such as organic matter decomposition and nutrient cycling, thus supporting the elementary activities linking above- and below-ground communities (Delgado-Baquerizo et al., 2016). However, interestingly, our results showed that microbial network structure, rather than plant and microbial community diversity, was the primary factor in driving multifunctionality, which was in agreement with the results of Zhai et al. (2024).

As a predictor of network complexity, microbial species interactions (edge number) have been considered to be a vital factor in regulating multifunctionality (Wagg et al., 2019), which is mainly affected by precipitation patterns change. Several lines of evidence confirm that microbial interactions are strengthened with higher precipitation, generating a more complex network. However, with the increase in annual precipitation, microbial network complexity declined, further enhancing ecosystem multifunctionality in the present study. Similarity, ecosystem, soil, N cycle, and P cycle multifunctionality were negatively associated with negative edge numbers under changes in precipitation (Figure 4), indicating that increased precipitation could strengthen multifunctionality by reducing microbial species competition. The wetter soil (waterlogging before the sampling in 2017) may destroy the microbial living environment, thus causing a decline in the robbing of nutrient resources by the microbial community, resulting in an increase in ecosystem and nutrient cycle functions. However, we know less about the contribution of bacteria and fungi in regulating microbial species interactions, necessitating further studies in the future.

## 5 Conclusion

Our findings suggested that multidiversity was mainly affected by increased precipitation, while multifunctionality was largely responsive to inter-annual variations in precipitation. Increased precipitation significantly reduced bacterial diversity and multidiversity, and a higher annual precipitation resulted in higher ecosystem, soil, N cycle, and P cycle multifunctionality. Our results also found that multidiversity and multifunctionality were decoupling, but microbial network structure mediated the response of multifunctionality to increased precipitation under different annual precipitation amount. In contrast to cooperative interactions, competitive interactions among microbial species determine multifunctionality. These results demonstrate that not only multidiversity but also the interactions between the species should be included in models to accurately evaluate the impact of changes in precipitation patterns on multifunctionality.

## Data availability statement

The datasets presented in this study can be found in online repositories. The names of the repository/repositories and accession number(s) can be found below: https://www.ncbi.nlm.nih.gov/, PRJNA545812.

## Author contributions

XuecY: Conceptualization, Data curation, Formal analysis, Funding acquisition, Investigation, Visualization, Writing—original draft, Writing—review & editing. WSo: Formal analysis, Visualization, Writing—review & editing. XueY: Formal analysis, Visualization, Writing—review & editing. TY: Investigation, Writing—review & editing. WB: Visualization, Writing—review & editing. CW: Investigation, Writing—review & editing. JL: Investigation, Writing—review & editing. SZ: Investigation, Writing—review & editing. QJ: Investigation, Writing—review & editing. LL: Supervision, Writing—review & editing. WSu: Conceptualization, Funding acquisition, Project administration, Supervision, Writing—original draft, Writing—review & editing.
