# Supplementary material for: Microbial network structure, not plant and microbial community diversity, regulates multifunctionality under increased precipitation in a cold steppe
Source: Front Microbiol. 2024 Jan 12;14:1349747. doi: 10.3389/fmicb.2023.1349747 (PMC10814129; doi:10.3389/fmicb.2023.1349747)
Supplement: Supplementary file 1 [file Data_Sheet_1.docx]

**Supplementary** **Material**

**Microbial network** **structure, not plant and microbial community diversity, regulates multifunctionality under increased precipitation in a cold steppe**

Xuechen Yang^1, 2^, Wenzheng Song^2^, Xue Yang^2, 3^, Tianxue Yang^2^, Wenqing Bao^1^, Chengliang Wang^2^, Junqin Li^2^, Shangzhi Zhong^2, 4^, Qi Jiang^2, 5^, Lu-Jun Li^1^ and Wei Sun^2, *^

^1^ State Key Laboratory of Black Soils Conservation and Utilization, Northeast Institute of Geography and Agroecology, Chinese Academy of Sciences, Harbin 150081, China.

^2^ Institute of Grassland Science, Key Laboratory of Vegetation Ecology of the Ministry of Education, Jilin Songnen Grassland Ecosystem National Observation and Research Station, Northeast Normal University, Changchun 130024, China.

^3^ School of Civil Engineering and Transportation, Northeast Forestry University, Harbin 150040, China.

^4^ Grassland Agri-Husbandry Research Center, College of Grassland Science, Qingdao Agricultural University, Qingdao 255109, China.

^5^ No. Fifteen Senior High School of Mudanjiang, Mudanjiang 157000, China.

***Correspondence: Wei Sun**

Institute of Grassland Science, Northeast Normal University

Changchun, Jilin 130024, China

Tel.: +86 431 8509 8187 Email: [sunwei@nenu.edu.cn](mailto:sunwei@nenu.edu.cn)

**Supplementary Table 1.** The PCR primers used for quantitative real-time PCR.

| Target gene  (Reference strain) | Primer | Primer sequence  (5’-3’) | Product size (bp) |
| --- | --- | --- | --- |
| Archaeal ammonium monooxygenase; *amoA* | CrenamoA23F  CrenamoA616R | ATGGTCTGGCTWAGACG  GCCATCCATCTGTATGTCCA | 629 |
| Bacterial ammonium monooxygenase; *amoA* | CTO189f A/B  CTO189fC  RT1r | GGAGRAAAGCAGGGGATCG  GGAGGAAAGTAGGGGATCG  CGTCCTCTCAGACCARCT | 115 |
| Bacterial 16S rRNA | 515-F  806-R | GTGCCAGCMGCCGCGGTAA  GGACTACHVGGGTWTCTAAT | 292 |
| Fungal ITS1 | ITS-1F  ITS-2R | CTTGGTCATTTAGAGGAAGTAA  GCTGCGTTCTTCATCGATGC | 272 |

Note: CTO189f A/B and CTO189fC were used in a 2:1 mixture.

**Supplementary Table 2.** Ecosystem function parameters across the increased precipitation gradient in 2016 and 2017. Values are mean ± SE (n = 4).

| **Year** | **Variable** | **I0** | **I30** | **I50** |
| --- | --- | --- | --- | --- |
| **2016** | PLC (g kg^−1^) | 458.4 ± 2.9 | 459.6 ± 3.2 | 453.3 ± 2.1 |
|  | PLN (g kg^−1^) | 17.6 ± 0.5 | 18.0 ± 1.7 | 17.2 ± 0.8 |
|  | BB (nmol g ^−1^) | 6.7 ± 0.5 | 6.0 ± 0.5 | 5.7 ± 0.6 |
|  | FB (nmol g ^−1^) | 1.2 ± 0.09 | 1.0 ± 0.11 | 1.0 ± 0.14 |
|  | DOC (mg kg^−1^) | 528.0 ± 132.1 | 547.4 ± 95.2 | 470.7 ± 201.7 |
|  | Net N min (mg kg^−1^ d^−1^) | 1.1 ± 0.5 | 1.3 ± 0.4 | 0.8 ± 0.3 |
|  | Pot N min (mg kg^−1^ d^−1^) | 0.25 ± 0.16 | 0.83 ± 0.11 | 0.29 ± 0.18 |
|  | AOA (copies g^−1^) | 2529.8 ± 1140.2 | 1148.3 ± 303.4 | 1570.2 ± 562.1 |
|  | AOB (copies g^−1^) | 4533.3 ± 523.9 | 4437.1 ± 532.4 | 4596.7 ± 560.5 |
|  | AP (mg kg^−1^) | 2.8 ± 0.3 | 2.8 ± 1.0 | 1.7 ± 0.5 |
|  | ALP (nmol g^−1^ h^−1^) | 38.3 ± 7.7 | 27.8 ± 3.5 | 18.3 ± 4.0 |
|  |  |  |  |  |
| **2017** | PLC (g kg^−1^) | 431.5 ± 0.7 | 441.7 ± 1.7 | 437.0 ± 1.0 |
|  | PLN (g kg^−1^) | 18.4 ± 0.8 | 20.9 ± 0.8 | 16.6 ± 1.6 |
|  | BB (nmol g ^−1^) | 6.4 ± 0.4 | 5.5 ± 0.7 | 6.4 ± 0.8 |
|  | FB (nmol g ^−1^) | 1.6 ± 0.18 | 1.2 ± 0.14 | 1.5 ± 0.19 |
|  | DOC (mg kg^−1^) | 397.3 ± 142.2 | 260.0 ± 133.9 | 516.7 ± 190.8 |
|  | Net N min (mg kg^−1^ d^−1^) | 0.6 ± 0.2 | 1.2 ± 0.2 | 0.6 ± 0.3 |
|  | Pot N min (mg kg^−1^ d^−1^) | 1.95 ± 0.27 | 1.79 ± 0.24 | 1.65 ± 0.32 |
|  | AOA (copies g^−1^) | (2.73 ± 0.97)×10^6^ | (2.56 ± 0.88)×10^6^ | (2.45 ± 0.78)×10^6^ |
|  | AOB (copies g^−1^) | 3572.9 ± 515.5 | 3370.1 ± 429.4 | 3835.6 ± 739.3 |
|  | AP (mg kg^−1^) | 4.5 ± 0.5 | 4.7 ± 0.4 | 4.8 ± 0.8 |
|  | ALP (nmol g^−1^ h^−1^) | 102.3 ± 8.2 | 97.3 ± 6.5 | 112.7 ± 16.7 |

Abbreviations: PLC, plant community leaf C content; PLN, plant community leaf N content; BB, bacterial biomass; FB, fungal biomass; DOC, soil dissolved organic C content; Net N min, net N mineralization rate; Pot N min, potential N mineralization rate; AOA, ammonia-oxidizing archaea abundance; AOB, ammonia-oxidizing bacteria abundance; AP, soil available phosphorus content; ALP, alkaline phosphatase.

**Supplementary Table 3.** Results (*F-values*) of linear mixed-effects models evaluating the effects of increased precipitation (IP), experimental year (Y), and their interactive effects on ecosystem function parameters (n = 4).

| Treatment | *df* | PLC | PLN | BB | FB | DOC | Net N min | Pot N min | AOA | AOB | AP | ALP |
| --- | --- | --- | --- | --- | --- | --- | --- | --- | --- | --- | --- | --- |
| IP | 2 | 6.48** | 3.83* | 0.967 | 2.094 | 0.177 | 0.177 | 1.84 | 0.03 | 0.17 | 0.372 | 0.388 |
| Y | 1 | 195.17*** | 1.81 | 0.001 | 10.089** | 0.977 | 0.770 | 83.97*** | 25.81*** | 4.42 | 17.778*** | 109.430*** |
| IP × Y | 2 | 5.15* | 1.90 | 0.659 | 0.675 | 0.588 | 0.157 | 2.16 | 0.03 | 0.04 | 0.662 | 1.655 |

Abbreviations: PLC, plant community leaf C content; PLN, plant community leaf N content; BB, bacterial biomass; FB, fungal biomass; DOC, soil dissolved organic C content; Net N min, net N mineralization rate; Pot N min, potential N mineralization rate; AOA, ammonia-oxidizing archaea abundance; AOB, ammonia-oxidizing bacteria abundance; AP, soil available phosphorus content; ALP, alkaline phosphatase.


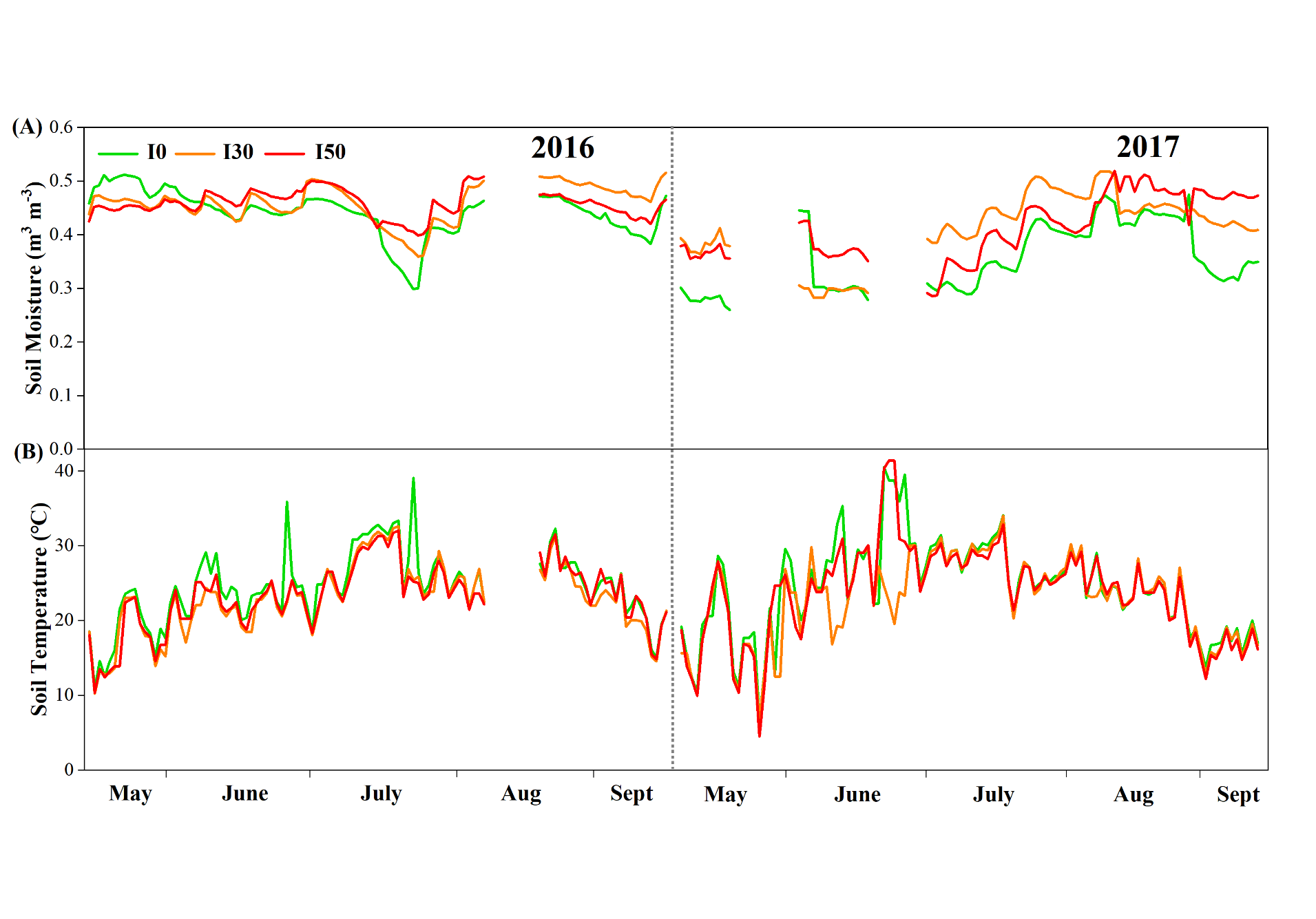


**Supplementary Fig. 1.** Variations in soil moisture and temperature at 0-30 cm soil depth for the increased precipitation treatments during the growing season of 2016 and 2017.
